# Imidazole-4-N-acetamide Derivatives as a Novel Scaffold for Selective Targeting of Cyclin Dependent Kinases

**DOI:** 10.3390/cancers15153766

**Published:** 2023-07-25

**Authors:** Polina Rusina, Erik Gandalipov, Yana Abdusheva, Maria Panova, Alexandra Burdenkova, Vasiliy Chaliy, Maria Brachs, Oleg Stroganov, Ksenia Guzeeva, Igor Svitanko, Alexander Shtil, Fedor Novikov

**Affiliations:** 1 Zelinsky Institute of Organic Chemistry, Russian Academy of Sciences, 47 Leninsky Avenue, 119991 Moscow, Russia; 2 Laboratory of Solution Chemistry and Advanced Materials Technologies, ITMO University, 9 Lomonosov Street, 191002 Saint Petersburg, Russia; 3 PHARMENTERPRISES LLC, Skolkovo Innovation Center, 42 (1) Bolshoi Blvd., 143026 Moscow, Russia; 4 Higher School of Economics, National Research University, 20 Myasnitskaya Street, 101000 Moscow, Russia; 5 Treamid Therapeutics GmbH, c/o CoLaborator (Bayer), Building S141, Muellerstraβe 178, 13353 Berlin, Germany; 6 BioMolTech Corp., Toronto, ON M2L 1L1, Canada; 7 Blokhin National Medical Research Center of Oncology, 24 Kashirskoye Shosse, 115522 Moscow, Russia; 8 Institute of Cyber Intelligence Systems, National Research Nuclear University MEPhI, 31 Kashirskoye Shosse, 115409 Moscow, Russia

**Keywords:** cyclin dependent kinases, CDK1, CDK2, CDK5, CDK9, NEQ, free energy calculation, selectivity prediction, molecular docking, molecular dynamics, tumor cell lines

## Abstract

**Simple Summary:**

General toxicity for the organism is a major drawback of anticancer drugs. Development of new generation chemotherapeutics requires the knowledge about macromolecules critical for tumor cell viability. Among these species (called therapeutic targets), the protein kinases are the established enzymes. However, the parts of protein kinases that are supposed to be targeted by drugs can be structurally similar. Therefore, it is difficult to design the compounds that selectively bind and inactivate individual kinases, especially if the proteins comprise the evolutionarily conserved families. In the present study, a set of advanced approaches of computational chemistry and biochemistry was used for the structure-based design of new compounds to inhibit cyclin-dependent protein kinases (CDK), the enzymes mechanistically implicated in tumor biology. We demonstrated the advantages of the non-equilibrium (NEQ) thermodynamics method for a time-efficacious, accurate, and informative prediction of CDK inhibitory properties of new compounds. Importantly, NEQ-based predictions correlated with experimental testing.

**Abstract:**

The rational design of cyclin-dependent protein kinase (CDK) inhibitors presumes the development of approaches for accurate prediction of selectivity and the activity of small molecular weight anticancer drug candidates. Aiming at attenuation of general toxicity of low selectivity compounds, we herein explored the new chemotype of imidazole-4-N-acetamide substituted derivatives of the pan-CDK inhibitor PHA-793887. Newly synthesized compounds **1**–**4** containing an aliphatic methyl group or aromatic radicals at the periphery of the scaffold were analyzed for the prediction of relative free energies of binding to CDK1, -2, -5, and -9 using a protocol based on non-equilibrium (NEQ) thermodynamics. This methodology allows for the demonstration of a good correlation between the calculated parameters of interaction of **1**–**4** with individual targets and the values of inhibitory potencies in in vitro kinase assays. We provide evidence in support of NEQ thermodynamics as a time sparing, precise, and productive approach for generating chemical inhibitors of clinically relevant anticancer targets.

## 1. Introduction

Cyclin-dependent kinases (CDKs) represent a family of vitally important cellular enzymes that, in complexes with respective cyclins and other protein partners, drive cell cycle progression and modulate gene transcription [1,2]. These properties made CDKs attractive anticancer drug targets. Indeed, inhibition of CDKs with small molecular weight compounds emerged as an area of extensive investigation at the crossroads of medicinal chemistry, cancer biology, and clinical practice. Nevertheless, efforts to design chemical CDK binders encounter serious methodological challenges, mainly the difficulties in predicting the selectivity of structurally similar targets. In particular, for CDK2 inhibitors, researchers deal with structural similarities between the catalytic domains of this enzyme and other CDKs, e.g., CDK1 and CDK9 in activated states [3]. A sizable portion (~30%) of non-selective inhibitors launched in the beginning of the millennium was terminated due to general toxicity [4]. Only recently, selective triple inhibitors of CDK2/4/6 were evaluated as a novel therapeutic approach for advanced or metastatic endocrine cancer in combination with conventional drugs [5,6,7]. 

We herein report the novel scaffold constructed on the basis of the pan-CDK inhibitor PHA-793887 for targeting individual CDKs [8]. In clinical trials, PHA-793887 caused an intolerable general toxicity manifested as gastrointestinal and neural system disorders, as well as severe dose-related hepatotoxicity [9]. In search of new potent inhibitors with selectivity to individual CDKs, we took advantage of the structure of PHA-793887 for the design of imidazole-4-N-acetamide-based CDK inhibitors [10]. Triazol- and pyrazol-containing compounds are known to evoke liver toxicity [11,12]. Furthermore, the pyrazole cycle was shown to mediate oxidative liver injury [13,14]. Because the hepatotoxicity of PHA-793887 may be attributed to the N-N fragment in the pyrazole cycle, we replaced this fragment with imidazole, whereas the isopropyl moiety in the ring remained unchanged [10]. 

Over the past decade, advances in computational techniques for predicting the free energies of the binding of compounds to their biological targets rendered this approach relevant for prioritizing the molecules for in silico design. The free energy perturbation (FEP) method uses equilibrium molecular dynamics (MD) to simulate ligand–protein complexes and allows for estimating the difference in the binding energy between two structurally similar ligands. Despite numerous successful applications, the classic FEP method requires significant computational resources to properly sample the ligand and protein conformations, a crucial factor for the accuracy of results. Recently, the time-efficacious non-equilibrium (NEQ) thermodynamics method was developed [15,16]. Its accuracy is comparable to FEP; moreover, the NEQ method effectively addresses the sampling issue implicit in FEP [17,18,19]. This problem, inherent to FEP, refers to the necessity for extensive, often computationally expensive, sampling of molecular states in order to achieve a robust estimation of free energy changes. Both methods were initially developed to predict the differences in the binding of free energy between ligands. Although these methods can theoretically predict the selectivity (i.e., the differential binding of the same ligand to individual proteins), their application in this specific context remains limited [20].

In the present work, we aimed at the prediction of the relative free energy of binding for newly synthesized imidazole-4-N-acetamide derivatives to their preferred target CDK2 and relative selectivity against CDK1, -5, and -9 using a protocol based on NEQ thermodynamics. This approach allowed us to predict relative binding energy of individual CDK inhibitors with a high degree of correlation between calculated and experimental values. The selected compounds demonstrated a micromolar cytotoxicity against a panel of human tumor cell lines, whereas non-malignant counterparts were spared. These results strengthen the validity of the NEQ approach as a promising tool in targeted drug discovery.

## 2. Materials and Methods

### 2.1. Chemicals

**Figure 1:**
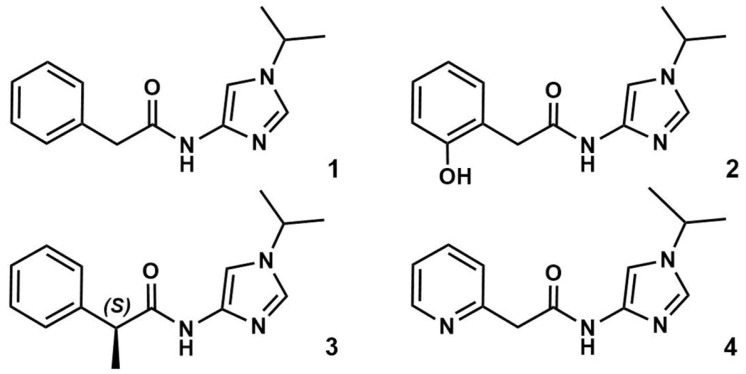
Structures of compounds **1**–**4**.

All reagents were obtained from Sigma-Aldrich unless specified otherwise. New imidazole-4-N-acetamide derivatives **1**–**4** (Figure 1) were synthesized as described [10]. Briefly, nitroimidazole was alkylated according to the published procedure [21]. The resulting nitro derivatives were reduced in isopropanol containing 2.2 equivalents of HCl (relative to the nitro derivative) in the presence of Pd on carbon. The obtained amino derivatives were acylated with respective phenylacetic acid derivatives using 2-(1H-benzotriazole-1-yl)-1,1,3,3-tetramethylaminium tetrafluoroborate as a coupling agent. Although **3** was obtained as a racemic mixture, only the S-isomer was used in the modeling because a similar scaffold demonstrated a CDK2 inhibitory potency [22].

### 2.2. In Silico Prediction of the Binding Affinity of Imidazole-4-N-acetamide Derivatives

**Figure 2:**
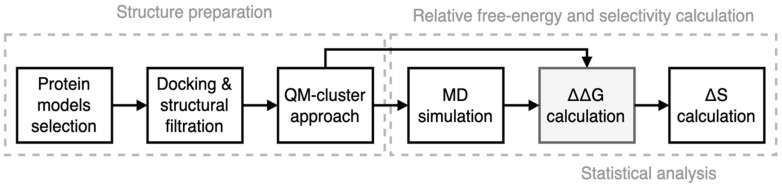
The workflow of the computational protocol.

We utilized the NEQ-based computational protocol for predicting the relative free energy (∆∆G) and selectivity (∆S) of new compounds (Figure 2). The optimal conformation of protein–ligand complexes was prepared using molecular docking, structural filtration, and a QM-based cluster approach (see below). The resulting systems were used for calculating ∆∆G and ∆S by the NEQ-based computational workflow pmx [23]. The accuracy of prediction was experimentally evaluated in in vitro kinase assays.

#### 2.2.1. Preparation of CDK–Ligand Complexes 

The X-ray crystal structures of CDK1, -2, -5, and -9 were obtained from the Protein Data Bank (Supplementary Materials, Section S1.1). Missing loops or other backbone fragments were constructed using PyMol 2.4.1 software [24] and refined by energy minimization in the OPLS force field using GROMACS 5.0 software [25]. The side chain reconstruction and protonation at pH 7.4 were performed with the Build Model software version 2112.1 [26,27] (Table S1). CDK–ligand complexes were constructed using molecular docking in combination with structural filtration [28] to select ligand conformations that form key hydrogen bonds with the kinase hinge region (Tables S2 and S3). The resulting conformations were in agreement with the structures of known CDK2 inhibitors that have similar scaffolds (Supplementary Materials, Section S1.2.1). We used the QM cluster approach based on the GFN2-xTB semi-empirical method to optimize target–ligand complexes and estimate the binding energy [29] (Supplementary Materials, Section S1.2.2). 

#### 2.2.2. NEQ Thermodynamics and Free Energy Calculations

We used NEQ free energy protocol pmx to predict the relative ligand binding affinity [18]. Ligand parametrization was performed using Antechamber [30] and ACPYPE [31] with the general amber force field [32]. Hybrid structures and topologies of ligands and complexes were created using the pmx algorithm [23,33]. We prepared the complexes of **1**–**4** for ΔΔG calculations, considering compound **1** as a reference for all transitions. Equilibrium molecular dynamic (MD) simulations were performed only for the physical end states for 10 ns in the GROMACS 5.0 software [25]. NEQ transitions for 50 ps were performed in the forward and backward directions using GROMACS [25]. The resulting free energy difference (∆∆G) was estimated using the pmx procedure [23] with the Jarzynski estimator (JAR) [34], Crooks Gaussian intersection (CGI) [35], and Bennet’s acceptance ratio (BAR) [36]. While JAR approximates the free energy difference by averaging many quick non-equilibrium changes [34], CGI calculates free energy differences using the intersection point of energy distributions obtained from forward and reverse transformations [35]. The BAR method enhances these estimations by optimal combination of forward and reverse data to calculate free energy differences [36]. The overview of calculation workflow is presented in Figure S2. 

### 2.3. In Vitro Kinase Assays

The inhibitory potencies of imidazole-4-N-acetamide derivatives against CDK2, -5, -7, and -9 were determined using the following protocol. The reaction mixture contained 0.1 mg/mL polyE4Y, the purified enzyme (0.1–0.7 pM), ^33^P ATP (1 μM), and the tested or reference (staurosporine) compounds at different concentrations. Mixtures were incubated for 2 h at 30 °C and spotted onto the ion exchange filter. The unbound phosphate was removed by extensive washing of filters in phosphoric acid. The IC_50_ and standard error (SE) values were obtained using GraphPad Prism 8 by log (inhibitor) vs. response (three parameters) curve analysis [37]. The IC_50_ values were converted to the inhibition constant K_i_ using the Cheng–Prusoff equation [38] based on Km values available in the literature [39,40,41,42]. Inhibition constants were converted to ∆∆G_(exp)_ values using the formula:

∆∆G_ij(exp)_ = ∆G_i_ − ∆G_j_ = −RTlnK_j_ − (−RTlnK_i_) = RTln(K_i_/K_j_), (1)

where ∆∆Gij_(exp)_ is the difference in free energy between the inhibitors i and j, ∆G_i_ and ∆G_j_ are Gibbs free energies of i and j, respectively. R is the universal gas constant, T is the absolute temperature in Kelvin; and K_i_ and K_j_ are the inhibition constants for i and j, respectively.

Experimental and total SE values were calculated using the formula similar to (1) from the symmetrical 95% confidence interval (CI) of IC_50_ values. 

### 2.4. Relative Selectivity Calculation

The approach developed by Albanese et al. was used to estimate the relative selectivity from ∆∆G values [20]. The difference in relative binding energies ΔS_ij_ can be used to predict the change in selectivity between the ligand i and a related ligand j in the target:

∆S_ij_ = S_i_ − S_j_  = (∆G_i_^CDKk^ − ∆G_i_^CDK2^) − (∆G_j_^CDKk^ − ∆G_j_^CDK2^) = ∆∆G_ij_^CDKk^ − ∆∆G_ij_^CDK2^, (2)

where CDK_k_ is CDK1, -5, or -9.

### 2.5. Anti-Proliferative Activity of New Imidazole-4-N-acetamide Derivatives

Human ovarian cancer (SKOV-3, OVCAR-3, OV-90, and UWB1.289) and neuroblastoma (IMR-32, SH-SY5Y, and Kelly) cell lines were obtained from the American Type Cancer Collection (Manassas, VA, USA). Human embryonic lung fibroblasts (HELF) and mesenchymal stem cells (MSC) were purchased from Biolot, Saint-Petersburg, Russia. Cells were cultured at 37 °C, 5% CO_2_ in a humidified atmosphere using the media and supplements recommended by the manufacturers. Experiments were conducted with cells in the logarithmic phase of growth. New imidazole-4-N-acetamide derivatives were reconstituted in dimethyl sulfoxide (vehicle) as 10 mM stock solutions. Serial dilutions in culture media were prepared immediately before experiments. Cells were plated overnight into 96-well plates (Corning, Corning, NY, USA; 5 × 10^3^ cells/well) and then treated with compounds **1**–**4** for 72 h at 37 °C, 5% CO_2_. After the completion of incubation, the medium was removed and cells were fixed with 10% trichloroacetic acid and stained with 0.4% solution of sulforhodamine B in 1% acetic acid. Plates were washed with 1% acetic acid, dried, and the bound sulforhodamine B was dissolved in 20 mM Tris-base solution. Absorbance at 570 nm was measured on a Tecan Spark spectrophotometer (Tecan; Männedorf, Switzerland). Cell viability was calculated as the absorbance in wells with the respective drug concentration divided by the absorbance in wells with the corresponding vehicle concentration multiplied by 100%.

### 2.6. Statistical Analysis 

The relationship between experimental and calculated differences of relative binding energies and the selectivity change was analyzed using the generalized Deming regression (version 1.4) in R programming language [43,44]. This method, unlike the conventional least squares regression, factors in uncertainties in both dependent and independent variables [45]. The 95% confidence interval (CI) of the slope coefficient was then computed. The presence of a statistically significant linear relationship between experimental and calculated data points was confirmed if the CI did not include zero.

## 3. Results

### 3.1. Preparation of Starting Target–Ligand Complexes and Calculations of Binding Energies

**Figure 3:**
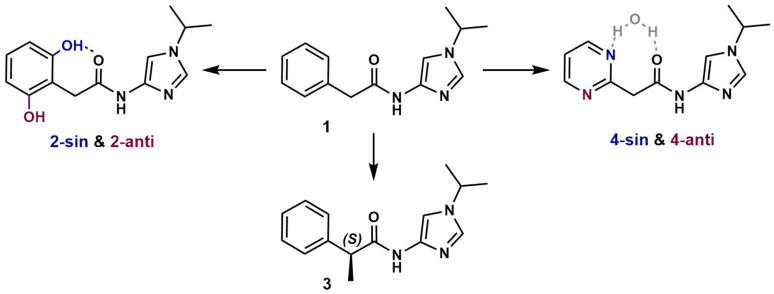
Ligand transitions used for ∆∆G calculations. Two alternative conformations of **2** and **4** are represented by colors.

An integrated approach was carefully designed to automatically sample the ligand conformations in the enzymes’ active sites. Molecular docking and structural filtration based on ‘hinge interaction’ (Supplementary Materials, Section S1.2.1) identified multiple conformations of **1**–**4** in CDK models. Using a QM-based approach, we found that, upon optimization, compounds **2** and **4** converged into two alternative conformations corresponding to different energy minima. Specifically, in **2**, the OH group can form an intramolecular hydrogen bond with the carbonyl oxygen atom or can be oriented in the opposite direction. We termed these conformations ‘sin-’ and ‘anti-’, respectively, for **2** and **4** (Figure 3).

In addition, we employed the QM-based cluster approach to calculate the target–ligand binding energies for sin- and anti- conformations. Our results indicate that sin**-2** and anti**-4** were preferred for CDK1, -2, and -9 (Tables S4 and S5, Figure S1). Since the actual ligand conformations in the active sites were unknown, we used all conformations to calculate ∆∆G by the NEQ method. This approach allowed us to avoid transitioning between local minima during MD simulations.

### 3.2. CDK Inhibitory Potency of New Imidazole-4-N-acetamide Derivatives

Compounds **1**–**4** have the ability to inhibit the activity of purified CDK1, -2, -5, and -9 in complexes with respective cyclins to phosphorylate the peptide substrates in a cell-free system (in vitro kinase assays). Table 1 shows that CDK2 in complexes with cyclin E was exceptionally sensitive to each compound, with IC_50_ values in the submicromolar range. The inhibitory profile of **3** was similar to that of **1** except for CDK1/cyclin E. Compound **2** demonstrated the highest inhibitory potency against all tested CDK/cyclin complexes. In contrast to **2**, compound **4** was several-fold less potent against CDK1/cyclin E and CDK5/p35.

### 3.3. Prediction of CDK Inhibitory Potency and Selectivity of Imidazole-4-N-acetamide Derivatives

According to NEQ calculations, the sin-**2** conformation was preferable for each examined CDK, whereas anti-**4** was favorable for CDK1 and -9 (Table S7). Results of QM and NEQ are largely consistent except for sin-**2** in CDK5 and sin-**4** for CDK2. Admittedly, the failure to account for dynamic influences, such as conformational focusing, limits the QM approach (Supplementary Materials, Section S2). To evaluate the accuracy of relative free energy predictions for **1** vs. **2** and **4**, we selected the conformation with the lowest ∆∆G values.

**Figure 4:**
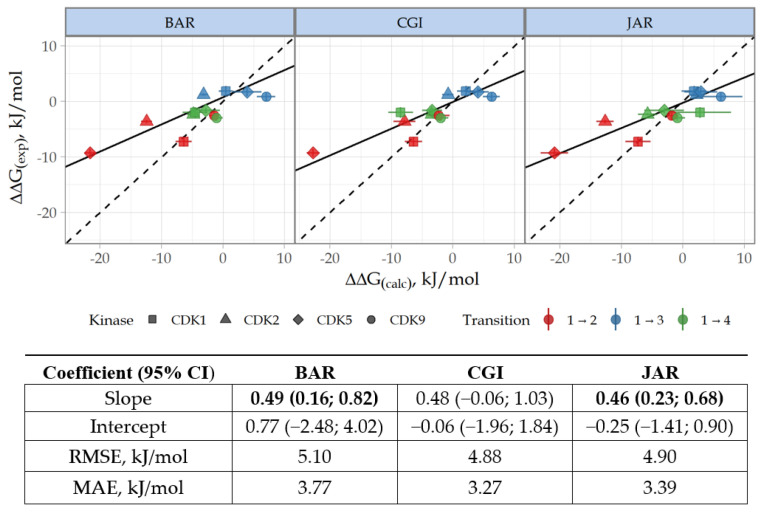
Correlation and Deming regression analyses for experimental ∆∆G(exp) and calculated ∆∆G(calc) relative free energy values. Data are presented for BAR, CGI, and JAR methods. The dashed line shows the identity. The slope coefficients of the linear regression with a statistically significant difference from 0 are shown in bold. RMSE, root mean square error; MAE, mean absolute error.

The NEQ-based predictions of relative free energy showed a good agreement with the kinase inhibitory potencies. The correlation coefficients R^2^ between experimental and calculated ∆∆G values were 0.67, 0.75, and 0.73 for BAR, CGI, and JAR methods, respectively. To account for experimental and calculated errors, we used Deming regression to fit the regression line assuming normally distributed errors in measurements of ∆∆G_(calc)_ and ∆∆G_(exp)_ (see Formula (1)). Apparently, the 95% CI values for Deming regression slopes were >0 for BAR and JAR methods, indicating statistically significant correlation (Figure 4).

**Figure 5:**
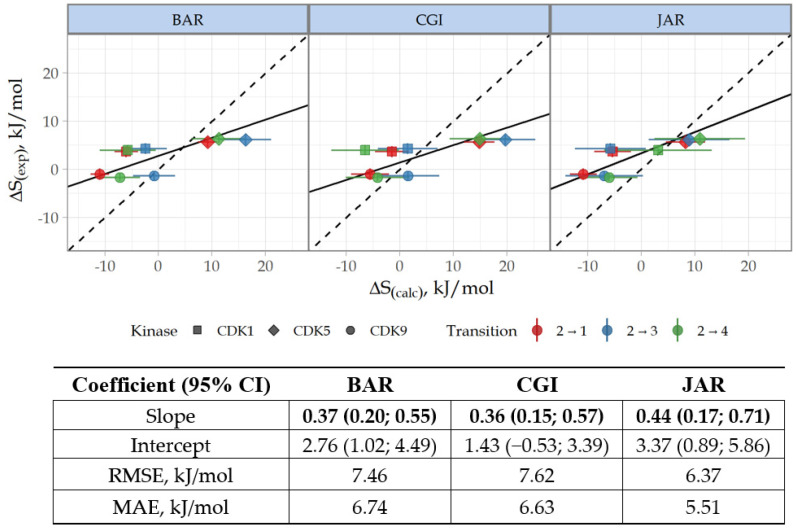
Correlation and Deming regression analyses for experimental ∆S(exp) and calculated ∆S(calc) relative selectivity values. See legend to Figure 4 for details.

The selectivity change ∆S between CDK2 ligands was calculated based on ∆∆G values according to the Formula (2), where k is CDK1, -5, or -9, i means compound **2**, and j means **1**, **3**, or **4** (Table S8). For clarity, the correlation of selectivity data is presented with reference to **2**, as this compound demonstrated no target selectivity. As expected, the reliability of ∆S prediction was lower since the ∆S metric includes the total error of the two ∆∆G values. The correlation coefficients R^2^ ∆S values were 0.52, 0.49, and 0.68 for BAR, CGI, and JAR methods, respectively. However, 95% CI for Deming regression slopes were greater than 0 for all methods (Figure 5). 

Our calculations demonstrate that, in general, NEQ methods provided consistent and accurate estimation of ∆∆G and ∆S. The mean absolute error (MAE) value for ∆∆G is 3.5 kJ/mol (equivalent to 0.83 kcal/mol), which fits the standard for ∆∆G estimation. In terms of selectivity, the JAR method performed unexpectedly better than the typically more precise CGI and BAR. The accuracy of ∆S prediction obtained by JAR is similar to that observed in relative FEP, for which the accuracy of ∆S prediction is 5.9 kJ/mol (1.4 kcal/mol). Nevertheless, we admit that the limitation stemming from the small sample size should be kept in mind for interpretation.

MD analysis revealed the reasons for superior inhibitory efficacy of compound **2**, over **1** and **3**, towards all examined CDKs (Table 1). In particular, the biologically active conformation of **2** is stabilized by a strong intramolecular hydrogen bond, while target binding by **1** and **3** requires an additional energy. We suggest that the H bond formation in solution decreases the conformational focusing, i.e., the energy required to convert all conformers in solution into the binding conformation [46]. Notably, compound **4**, in which an intramolecular bond was formed through water molecule, exhibited similar behavior (for more details see Supplementary Materials Section S2, Figures S3 and S4). 

Furthermore, the higher inhibitory potency of **2**, in comparison to **4**, towards CDK1 and -5 is likely due to π-stacking interactions of the aromatic ring with Lys89 (Table S6). Using electrostatic potential calculations, we demonstrated that the charge distribution in the phenol moiety promotes π-stacking and enhances the binding of **2** to CDK1 and -5 compared to benzene (compounds **1** and **3**) and pyridine (compound **4**) moieties (for more details see Supplementary Materials Section S3, Figure S5).

### 3.4. Cytotoxicity of Novel Imidazole-4-N-acetamide Derivatives

Compounds **2** and **4** were the two most potent CDK inhibitors in in vitro kinase assays (Table 1). At submicromolar concentrations, **2** inhibited all tested CDKs in complexes with cognate cyclin partners, whereas **4** preferentially targeted CDK2/cyclin E and CDK9/cyclin K. We were interested in whether the potency in cell-free systems can be translated into the ability to induce the death of human tumor cell lines. Our panel included ovarian cancer and neuroblastoma, the tumor types in which CDKs were validated as therapeutic targets [47]. Both **2** and **4** were cytotoxic at low micromolar concentrations in all tested tumor cell lines, with SH-SY5Y neuroblastoma being an exception. Importantly, non-malignant human HELF and MSC cells were not affected even after 72 h of exposure (Table 2).

## 4. Discussion

This study shows that the computational protocol based on methods of NEQ thermodynamics is efficient for the optimization of the binding affinity and selectivity of small molecular weight compounds as CDK/cyclin inhibitors. The use of Deming regression confirmed the statistical significance of the correlation between calculated and experimental ∆∆G and ∆S values. All NEQ methods showed comparable accuracy of ∆∆G and ∆S estimation, with the JAR method being a potential alternative to the relative FEP method in predicting ∆S. The observed high accuracy is important because the calculation was performed without core NEQ protocol adjustment and took ~40% less time than the enhanced FEP calculations [20].

Addressing the problem of extensive sampling of multiple local minima, we used a comprehensive workflow for preparing starting target–ligand complexes based on molecular docking, structural filtration, and QM. We used the semi-empirical QM cluster approach [29] to optimize alternative ligand conformations in different local energy minima identified by molecular docking. For each local minimum of the ligand conformation, we used the optimized structures for MD simulations and NEQ. By utilizing multiple conformations, we constrained the sampling to the region of the local minimum, thereby shortening the time of MD simulations.

Importantly, the QM-based estimation of target–ligand binding energy allows for qualitatively assessing the biological activity (Supplementary Materials, Section S1.2.2) and is therefore pertinent to the initial prediction of the compound’s selectivity profile. Nevertheless, we anticipated that the MD-based relative free energy calculation would yield more rigorous affinity predictions. Additionally, MD analysis provides a qualitative explanation for differences in binding associated with structural modifications of ligands.

Finally, we demonstrated that **2** and **4** were cytotoxic for tumor types in which CDKs are being investigated as tentative therapeutic targets. The cytotoxicity was achieved at low micromolar concentrations of each compound. Importantly, non-malignant cells were not affected in the tested dose range, suggesting a safe application in a broad therapeutic window. Both compounds were similarly potent against a series of ovarian carcinoma and neuroblastoma cell lines except SH-SY5Y. Mechanisms of differential efficacy of **2** and **4** for individual cell lines remain to be elucidated. Our calculation and in vitro data indicated similar, although not identical, inhibitory profiles for compounds **2** and **4**, with compound **2** being less selective. The common targets for these agents were CDK2/cyclin E and CDK9/cyclin K (Table 1). One may surmise that inhibition of CDK1/cyclin E and CDK5/p35 complexes by **2** is tolerable, whereas targeting CDK2/cyclin E and/or CDK9/cyclin K is lethal. Moreover, targets of our new compounds may not be confined solely to the tested CDKs. An in-depth study of molecular ‘portraits’ of individual tumors is underway to evaluate the mechanisms of sensitivity of specific tumor types to the new chemotype.

## 5. Conclusions

Aiming at optimization of small molecular weight CDK inhibitors, this study established the protocol of sampling local minima of ligand conformations in the active sites of homologous enzymes for predicting the relative free energy of binding and ligand selectivity. This approach integrated the molecular docking, structural filtration, and QM-based clustering to prepare optimal protein–ligand complexes, which were then used to calculate ∆∆G and ∆S through the NEQ-based computational workflow. The developed procedure provided the rationale for changes in the activity and specificity in the course of transitions between imidazole-4-N-acetamide derivatives. The predicted CDK inhibitory profiles as well as the cytotoxic potency against tumor cells without affecting non-malignant counterparts prove the potential of the new scaffold in antitumor drug design.

## Figures and Tables

**Table 1:** In vitro inhibitory activities of **1**–**4** and PHA-793887 [8] against purified CDK/cyclin complexes.

Target	Compound 1	Compound 2	Compound 3	Compound 4	PHA-793887
CDK1/cyclin E	14 (8–27)	0.72 (0.54–0.96)	30 (19–49)	6.2 (4.0–9.9)	0.060 (cyclin B)
CDK2/cyclin E	0.71 (0.64–0.80)	0.16 (0.13–0.19)	1.2 (1.0–1.3)	0.27 (0.24–0.31)	0.008
CDK5/p35	40 (27–59)	0.88 (0.65–1.19)	79 (49–126)	20 (16–25)	0.006 (p25)
CDK9/cyclin K	3.0 (1.7–5.3)	1.0 (0.7–1.5)	4.3 (3.0–6.1)	0.88 (0.83–0.94)	0.138 (cyclin T1)

Values are IC_50_ (µM) with 95% CI.

**Table 2:** Cytotoxicity of **2** and **4** against tumor and non-malignant cell lines.

Cell Line	Compound 2	Compound 4
SKOV-3	4.4 ± 1.2	3.3 ± 0.2
OVCAR-3	6.9 ± 0.5	5.8 ± 0.4
OV-90	3.9 ± 0.6	2.4 ± 0.3
UWB1.289	2.1 ± 0.2	1.7 ± 0.1
IMR-32	4.2 ± 0.1	3.2 ± 0.1
Kelly	8.6 ± 2.1	9.2 ± 2.4
SH-SY5Y	55.9 ± 3.7	57.8 ± 8.4
HELF	>100	>100
MSC	>100	>100

Shown are IC_50_ (µM; mean ± SD of four experiments).

## Data Availability

The data presented in this study are available in this article.

## Supplementary Materials

The following supporting information can be downloaded at: https://www.mdpi.com/article/10.3390/cancers15153766/s1. Table S1. Characteristics of CDK models; Table S2. CDK2 binders with scaffolds similar to **1**–**4**; Table S3. Characteristics of target-ligand complexes; Table S4. QM-based ΔΔG calculations for forward reactions. Bold fonts denote optimal conformations; Table S5. QM-based ΔΔG calculations for reverse reactions. Bold fonts denote optimal conformations; Table S6. Key interactions for OH group in **2** and N atom in **4** in complexes with CDK1, -2, -5, and- 9 obtained by MD trajectory analysis; Table S7. ∆∆G calculations. Green indicates ddG values that differ significantly from zero; gray indicates an unreliable difference from zero. Red indicates an incorrect forecast. Bold in the Transition column denotes conformations that correspond to energy minima; Table S8. ∆S calculations. Color codes: green, ∆S differs significantly from zero; gray, difference from zero is not reliable. Pink, incorrect prediction. Bold in the Transition column denotes conformations that correspond to energy minima; Figure S1. Correlation analyses for experimental and calculated ratios of inhibition constants in the log scale. The dashed line shows the identity; Figure S2. The computational algorithm for ∆∆G calculation based on pmx protocol [8]; Figure S3. Correlation between relative electronic energy and OCCC angles for **1**–**4**; Figure S4. The contour map of 2D potential energy surfaces of **1** (top) and **3** (bottom) with overlaid points based on MD; Figure S5. Total electrostatic potentials for arenes. References [48,49,50,51,52,53,54,55,56,57,58,59] are cited in the supplementary materials.

## References

[1] Malumbres M., Barbacid M. (2009). Cell cycle, CDKs and cancer: A changing paradigm. Nat. Rev. Cancer.

[2] Basu S., Greenwood J., Jones A.W., Nurse P. (2022). Core control principles of the eukaryotic cell cycle. Nature.

[3] Echalier A., Hole A.J., Lolli G., Endicott J.A., Noble M.E.M. (2014). An inhibitor’s-eye view of the ATP-binding site of CDKs in different regulatory states. ACS Chem. Biol..

[4] Whittaker S.R., Mallinger A., Workman P., Clarke P.A. (2017). Inhibitors of cyclin-dependent kinases as cancer therapeutics. Pharmacol. Ther..

[5] Rusina P.V., Lisov A.A., Denisova A.A., Gandalipov E.R., Novikov F.N., Shtil A.A. (2022). Clinical CDK2 Inhibitors: Trends To Selectivity and Efficacy. Recent Pat. Anticancer Drug Discov..

[6] Koirala N., Dey N., Aske J., De P. (2022). Targeting Cell Cycle Progression in HER2+ Breast Cancer: An Emerging Treatment Opportunity. Int. J. Mol. Sci..

[7] Hassan M.A.-K., Ates-Alagoz Z. (2022). Cyclin-Dependent Kinase 4/6 Inhibitors Against Breast Cancer. Mini Rev. Med. Chem..

[8] Brasca M.G., Albanese C., Alzani R., Amici R., Avanzi N., Ballinari D., Bischoff J., Borghi D., Casale E., Croci V. (2010). Optimization of 6,6-dimethyl pyrrolo[3,4-c]pyrazoles: Identification of PHA-793887, a potent CDK inhibitor suitable for intravenous dosing. Bioorg. Med. Chem..

[9] Massard C., Soria J.-C., Anthoney D.A., Proctor A., Scaburri A., Pacciarini M.A., Laffranchi B., Pellizzoni C., Kroemer G., Armand J.-P. (2011). A first in man, phase I dose-escalation study of PHA-793887, an inhibitor of multiple cyclin-dependent kinases (CDK2, 1 and 4) reveals unexpected hepatotoxicity in patients with solid tumors. Cell Cycle.

[10] Rusina P.V., Titov I.Y., Panova M.V., Stroylov V.S., Abdyusheva Y.R., Murlatova E.Y., Svitanko I.V., Novikov F.N. (2020). Modeling of novel CDK7 inhibitors activity by molecular dynamics and free energy perturbation methods. Mendeleev Commun..

[11] Albrecht W. (2019). Highlight report: Hepatotoxicity of triazole fungicides. Arch. Toxicol..

[12] Kharb R., Sharma P.C., Yar M.S. (2011). Pharmacological significance of triazole scaffold. J. Enzym. Inhib. Med. Chem..

[13] Lu Y., Gong P., Cederbaum A.I. (2008). Pyrazole induced oxidative liver injury independent of CYP2E1/2A5 induction due to Nrf2 deficiency. Toxicology.

[14] Wang X., Wu D., Yang L., Cederbaum A.I. (2011). Hepatotoxicity mediated by pyrazole (cytochrome P450 2E1) plus tumor necrosis factor alpha treatment occurs in c-Jun N-terminal kinase 2^−/−^ but not in c-Jun N-terminal kinase 1^−/−^ mice. Hepatology.

[15] Li P., Jia X., Wang M., Mei Y. Comparison of Accuracy and Convergence Rate between Equilibrium and Nonequilibrium Alchemical Transformations for Calculation of Relative Binding Free Energy. http://cjcp.ustc.edu.cn/html/hxwlxb_cn/2017/6/cjcp1711204.htm.

[16] Breznik M., Ge Y., Bluck J.P., Briem H., Hahn D.F., Christ C.D., Mortier J., Mobley D.L., Meier K. (2023). Prioritizing small sets of molecules for synthesis through in-silico tools: A comparison of common ranking methods. ChemMedChem.

[17] Procacci P. (2021). Methodological uncertainties in drug-receptor binding free energy predictions based on classical molecular dynamics. Curr. Opin. Struct. Biol..

[18] Gapsys V., Pérez-Benito L., Aldeghi M., Seeliger D., van Vlijmen H., Tresadern G., de Groot B.L. (2020). Large scale relative protein ligand binding affinities using non-equilibrium alchemy. Chem. Sci..

[19] Gapsys V., Yildirim A., Aldeghi M., Khalak Y., van der Spoel D., de Groot B.L. (2021). Accurate absolute free energies for ligand–protein binding based on non-equilibrium approaches. Commun. Chem..

[20] Albanese S.K., Chodera J.D., Volkamer A., Keng S., Abel R., Wang L. (2020). Is Structure-Based Drug Design Ready for Selectivity Optimization?. J. Chem. Inf. Model..

[21] Chacón Simon S., Wang F., Thomas L.R., Phan J., Zhao B., Olejniczak E.T., Macdonald J.D., Shaw J.G., Schlund C., Payne W. (2020). Discovery of WD Repeat-Containing Protein 5 (WDR5)–MYC Inhibitors Using Fragment-Based Methods and Structure-Based Design. J. Med. Chem..

[22] Pevarello P., Brasca M.G., Orsini P., Traquandi G., Longo A., Nesi M., Orzi F., Piutti C., Sansonna P., Varasi M. (2005). 3-Aminopyrazole Inhibitors of CDK2/Cyclin A as Antitumor Agents. 2. Lead Optimization. J. Med. Chem..

[23] Gapsys V., Michielssens S., Seeliger D., de Groot B.L. (2015). pmx: Automated protein structure and topology generation for alchemical perturbations. J. Comput. Chem..

[24] PyMOL | pymol.org. https://pymol.org/2/.

[25] Abraham M.J., Murtola T., Schulz R., Páll S., Smith J.C., Hess B., Lindahl E. (2015). GROMACS: High performance molecular simulations through multi-level parallelism from laptops to supercomputers. SoftwareX.

[26] Stroganov O.V., Novikov F.N., Stroylov V.S., Kulkov V., Chilov G.G. (2008). Lead finder: An approach to improve accuracy of protein-ligand docking, binding energy estimation, and virtual screening. J. Chem. Inf. Model..

[27] Stroganov O.V., Novikov F.N., Zeifman A.A., Stroylov V.S., Chilov G.G. (2011). TSAR, a new graph–theoretical approach to computational modeling of protein side-chain flexibility: Modeling of ionization properties of proteins. Proteins Struct. Funct. Bioinform..

[28] Novikov F.N., Stroylov V.S., Stroganov O.V., Chilov G.G. (2010). Improving performance of docking-based virtual screening by structural filtration. J. Mol. Model..

[29] Losev T.V., Gerasimov I.S., Panova M.V., Lisov A.A., Abdyusheva Y.R., Rusina P.V., Zaletskaya E., Stroganov O.V., Medvedev M.G., Novikov F.N. (2023). Quantum Mechanical-Cluster Approach to Solve the Bioisosteric Replacement Problem in Drug Design. J. Chem. Inf. Model..

[30] Wang J., Wang W., Kollman P.A., Case D.A. (2006). Automatic atom type and bond type perception in molecular mechanical calculations. J. Mol. Graph. Model..

[31] Sousa da Silva A.W., Vranken W.F. (2012). ACPYPE-AnteChamber PYthon Parser interfacE. BMC Res. Notes.

[32] Wang J., Wolf R.M., Caldwell J.W., Kollman P.A., Case D.A. (2004). Development and testing of a general amber force field. J. Comput. Chem..

[33] Gapsys V., de Groot B.L. (2017). pmx Webserver: A User Friendly Interface for Alchemistry. J. Chem. Inf. Model..

[34] Jarzynski C. (1997). Nonequilibrium Equality for Free Energy Differences. Phys. Rev. Lett..

[35] Crooks G.E. (1998). Nonequilibrium Measurements of Free Energy Differences for Microscopically Reversible Markovian Systems. J. Stat. Phys..

[36] Bennett C.H. (1976). Efficient estimation of free energy differences from Monte Carlo data. J. Comput. Phys..

[37] Home-GraphPad. https://www.graphpad.com/.

[38] Cheng Y., Prusoff W.H. (1973). Relationship between the inhibition constant (K1) and the concentration of inhibitor which causes 50 per cent inhibition (I50) of an enzymatic reaction. Biochem. Pharmacol..

[39] Kõivomägi M., Valk E., Venta R., Iofik A., Lepiku M., Morgan D.O., Loog M. (2011). Dynamics of Cdk1 Substrate Specificity during the Cell Cycle. Mol. Cell.

[40] Sitcheran R., Gupta P., Fisher P.B., Baldwin A.S. (2005). Positive and negative regulation of EAAT2 by NF-κB: A role for N-myc in TNFα-controlled repression. EMBO J..

[41] Hashiguchi M., Saito T., Hisanaga S., Hashiguchi T. (2002). Truncation of CDK5 Activator p35 Induces Intensive Phosphorylation of Ser202/Thr205 of Human Tau. J. Biol. Chem..

[42] Baumli S., Hole A.J., Wang L.-Z., Noble M.E.M., Endicott J.A. (2012). The CDK9 Tail Determines the Reaction Pathway of Positive Transcription Elongation Factor b. Structure.

[43] Package Deming-CRAN. https://CRAN.R-project.org/package=deming.

[44] R: The R Project for Statistical Computing. https://www.r-project.org/.

[45] Martin R.F. (2000). General Deming Regression for Estimating Systematic Bias and Its Confidence Interval in Method-Comparison Studies. Clin. Chem..

[46] Zeifman A.A., Stroylov V.V., Novikov F.N., Stroganov O.V., Kulkov V., Chilov G.G. (2013). Alchemical FEP Calculations of Ligand Conformer Focusing in Explicit Solvent. J. Chem. Theory Comput..

[47] Łukasik P., Załuski M., Gutowska I. (2021). Cyclin-Dependent Kinases (CDK) and Their Role in Diseases Development—Review. Int. J. Mol. Sci..

[48] Martin M.P., Endicott J.A., Noble M.E.M. (2017). Structure-based discovery and development of cyclin-dependent protein kinase inhibitors. Essays Biochem.

[49] Lee J., Choi H., Kim K.-H., Jeong S., Park J.-W., Baek C.-S., Lee S.-H. (2008). Synthesis and biological evaluation of 3,5-diaminoindazoles as cyclin-dependent kinase inhibitors. Bioorganic Med. Chem. Lett..

[50] Helal C.J., Kang Z., Lucas J.C., Gant T., Ahlijanian M.K., Schachter J.B., Richter K.E.G., Cook J.M., Menniti F.S., Kelly K. (2009). Potent and cellularly active 4-aminoimidazole inhibitors of cyclin-dependent kinase 5/p25 for the treatment of Alzheimer’s disease. Bioorganic Med. Chem. Lett..

[51] Adamo C., Barone V. (1999). Toward reliable density functional methods without adjustable parameters: The PBE0 model. J. Chem. Phys..

[52] Grimme S., Antony J., Ehrlich S., Krieg H. (2010). A consistent and accurate ab initio parametrization of density functional dispersion correction (DFT-D) for the 94 elements H-Pu. J. Chem. Phys..

[53] Grimme S., Ehrlich S., Goerigk L. (2011). Effect of the damping function in dispersion corrected density functional theory. J. Comput. Chem..

[54] Weigend F., Ahlrichs R. (2005). Balanced basis sets of split valence, triple zeta valence and quadruple zeta valence quality for H to Rn: Design and assessment of accuracy. Phys. Chem. Chem. Phys..

[55] Frisch M.J., Trucks G.W., Schlegel H.B., Scuseria G.E., Robb M.A., Cheeseman J.R., Scalmani G., Barone V., Petersson G.A., Nakatsuji H. (2016). Gaussian 16 Revision A.03.

[56] Mennucci B. (2012). Polarizable continuum model. WIREs Comput. Mol. Sci..

[57] Luchini G., Alegre-Requena J.V., Guan Y., Funes-Ardoiz I., Paton R.S. (2019). GoodVibes. https://anaconda.org/patonlab/goodvibes.

[58] Zhang J., Lu T. (2021). Efficient evaluation of electrostatic potential with computerized optimized code. Phys. Chem. Chem. Phys..

[59] Lu T., Chen F. (2012). Multiwfn: A multifunctional wavefunction analyzer. J. Comput. Chem..

