# Supplementary material for: Imidazole-4-N-acetamide Derivatives as a Novel Scaffold for Selective Targeting of Cyclin Dependent Kinases
Source: Cancers (Basel). 2023 Jul 25;15(15):3766. doi: 10.3390/cancers15153766 (PMC10417023; doi:10.3390/cancers15153766)
Supplement: Supplementary file 1 [file cancers-15-03766-s001.zip › cancers-2454736-supplementary.pdf]

Imidazole-4-N-acetamide Derivatives as a Novel Scaffold for Preferential Targeting of Cyclin Dependent Kinases

Polina Rusina [1], Erik Gandalipov [2,3], Yana Abdusheva [1,3,4], Maria Panova [1,3], Alexandra Burdenkova [1,4], Vasiliy Chaliy [1], Maria Brachs [5], Oleg Stroganov [6], Ksenia Guzeeva [4], Igor Svitanko [1,4], Alexander Shtil [7,8,*] and Fedor Novikov [1,3,4,*]

[1] Zelinsky Institute of Organic Chemistry, Russian Academy of Sciences, 47 Leninsky Avenue, 119991 Moscow, Russia
[2] Laboratory of Solution Chemistry and Advanced Materials Technologies, ITMO University, 9 Lomonosov Street, 191002 Saint-Petersburg, Russia
[3] PHARMENTERPRISES LLC, Skolkovo Innovation Center, 42 (1) Bolshoi Blvd., 143026 Moscow, Russia
[4] Higher School of Economics, National Research University, 20 Myasnitskaya Street, 101000 Moscow, Russia
[5] Treamid Therapeutics GmbH, c/o CoLaborator (Bayer), Building S141, Muellerstraβe 178, 13353 Berlin, Germany
[6] BioMolTech Corp., Toronto, ON M2L 1L1, Canada
[7] Blokhin National Medical Research Center of Oncology, 24 Kashirskoye Shosse, 115522 Moscow, Russia
[8] Institute of Cyber Intelligence Systems, National Research Nuclear University MEPhI, 31 Kashirskoye Shosse, 115409 Moscow, Russia
* Correspondence: shtilaa@yahoo.com (A.S.); fnovikov@pharmenterprises.ru (F.N.)

1 *In silico* prediction of the binding affinity of imidazole-4-N-acetamide derivatives

1.1 Preparation of the protein model

The structures of CDKs were obtained from Protein Data Bank (PDB), UniProt accession numbers P06493, P24941, Q00535, and P50750 for CDK1, CDK2, CDK5 and CDK9, respectively. The proteins without the ligand or cyclin partners, as well as the structures crystallized under pH < 6 or under an unknown pH, were excluded from all CDK-inhibitor complexes in PDB. The PDB IDs of final structures were 6GU3 (CDK1), 7KJS (CDK2), 1UNL (CDK5), and 4BCH (CDK9). The remaining protein structures were automatically prepared by side chain reconstruction and protonation, assigning the amino acid ionization states at pH = 7.4 using the Build Model module in the Lead Finder package [26, 27] (Table S1).

Table S1. Characteristics of CDK models.

Protein	PDB ID	pH	Net charge pH = 7.4	Net charge pH = 7.4 prepared
CDK1	6GU3	6.7	+1.408	+2
CDK2	7KJS	6.0	+3.290	+5
CDK5	1UNL	7.0	+0.564	+3
CDK9	4BCH	6.2	+6.696	+4

1.2 Construction and optimization of CDK-ligand complex

1.2.1 Molecular docking and structural filtration

Protein-ligand structures were prepared using molecular docking. The optimized structures of compounds **2-4** were docked into the sets of X-ray crystallographic structures of CDK1, -2, -5, and 9 (10 times for each target) using Lead Finder software package version 2112.1 [26, 27]. Then structural filtration [28] was used for every CDK-inhibitor set to assess the validity of structures by key hinge interactions (Leu-83 for CDK1 and -2, Cys-83 for CDK5, and Cys-106 for CDK9) [48]. Final complexes were chosen by maximum docking score between the structures subjected to filtration. Structures with **1** were prepared based on the corresponding complexes with **2-4**. Dihedral angles O=C-C-C of resulting ligand conformations were compared with known structures of CDK2 inhibitors that have similar scaffolds (Tables S2, S3).

Table S2. CDK2 binders with scaffolds similar to **1-4**.

PDB	Resolution. Å	Structure of reference ligand	IC_{50}. nM	Dihedral angle O=C-C-C	Link
2BPM	2.30	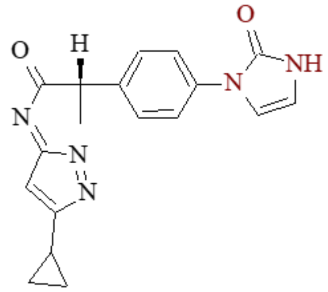	2	72.6	[48]
2R64	1.90	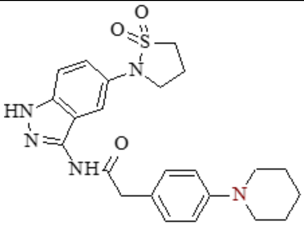	30	59.6	[22]
3IG7	2.20	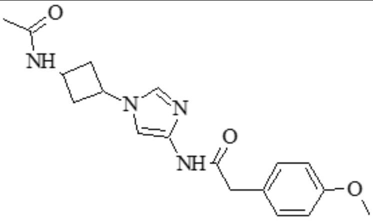	63/9	70.8	[49]
3IGG	2.30	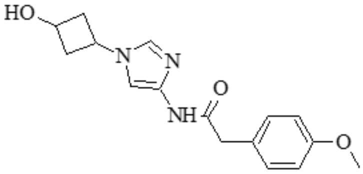	66.5/95	87.3	[50]
4EK6	2.00	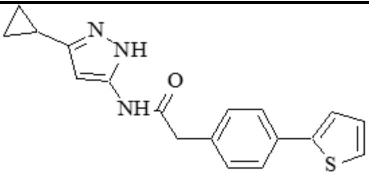	3	81.8	10.2210/pdb4EK6/pdb
4FKI	2.60	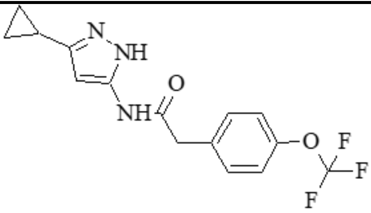	67	85.0	10.2210/pdb4FKI/pdb
4FKJ	2.60	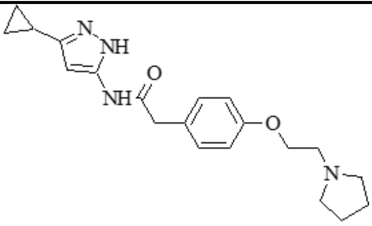	-	81.6	10.2210/pdb4FKJ/pdb

Table S3. Characteristics of target-ligand complexes.

Target	Transition	Dihedral angle O=C-C-C for complexes with compound 1	Dihedral angle O=C-C-C for complexes with compound 2
CDK1	1 → 2 sin	77.6	78.2
CDK1	1 → 2 anti	88.8	94.8
CDK1	1 → 3	78.5	87.3
CDK1	1 → 4 sin	77.7	78.4
CDK1	1 → 4 anti	76.7	72.7
CDK2	1 → 2 sin	84.1	86.7
CDK2	1 → 2 anti	76.0	67.8
CDK2	1 → 3	74.1	67.1
CDK2	1 → 4 sin	83.5	72.6
CDK2	1 → 4 anti	84.9	84.9
CDK5	1 → 2 sin	65.4	65.3
CDK5	1 → 2 anti	75.4	72.9
CDK5	1 → 3	95.4	95.6
CDK5	1 → 4 sin	73.5	100.8
CDK5	1 → 4 anti	74.2	67.3
CDK9	1 → 2 sin	95.5	90.2
CDK9	1 → 2 anti	81.4	79.9
CDK9	1 → 3	82.0	81.4
CDK9	1 → 4 sin	78.2	76.4
CDK9	1 → 4 anti	77.0	71.5

1.2.2 QM optimization and relative binding energy calculations

The structures of proteins and selected ligand poses from the previous step were used for model optimization. This involved taking the ligand and kinase residues within 4−5 Å from the ligand, constructing a convex hull with additional penetrating residues and excluding all water molecules from the model. The positional restraints were used for α-carbon and associated hydrogen atoms. The sampling of the configuration space was performed using the semi-empirical QM method GFN2-xTB implemented in XTB 6.4.1 supplemented with the GBSA solvation model to obtain the global minimum. The lowest energy structure was then modified through atomic replacement and optimization. Single-point QM calculations were used to estimate the energy of unbound ligand in solution. The obtained ligand structure was then subjected to atomic replacement followed by single-point optimization to determine the relative binding energy between two ligands (Table S4). The resulting free energy difference $\Delta\Delta G_{ij}$ between ligand *i* and ligand *j* was calculated using the equation:

$$\Delta\Delta G_{ij} = \Delta G_j - \Delta G_i = \left(G_{complex_j} - G_{ligand_j}\right) - \left(G_{complex_i} - G_{ligand_i}\right).$$

where $G_{complex_j}$ is total energy including the energies of the protein, the ligand *j* and protein-ligand interaction expressed in Hartree energy; G_{ligand_j} is total energy of optimized unbound ligand *j* in solution expressed in Hartree energy; $G_{complex_i}$ is total energy including the energies of the protein, the ligand *i* and protein-ligand interaction expressed in Hartree energy; G_{ligand_i} is total energy of optimized unbound ligand *i* in solution expressed in Hartree energy.

The logarithmic form of the link between the free energy of reaction and the activity ratio was calculated as:

$$\Delta\Delta G_{ij} = \Delta G_j - \Delta G_i = -RT\ ln\ ln\ K_j\ - (-RT\ ln\ ln\ K_i\ ) = -RT\ ln\ ln\ \frac{K_j}{K_i}\ .$$

where K_i and K_j are inhibition constants.

The sensitivity analysis was performed to assess the reliability of QM data. Reverse transitions of the presented reactions were calculated (Table S5). The optimized structures of the complex *j* and the unbound ligand *j* were taken for reverse atomic replacement and subsequent QM optimization. Ratios of inhibition constants for the forward reaction were compared with the values obtained during the reverse reaction. Similar results for both transitions confirmed the reliability of calculations.

Table S4. QM-based ΔΔG calculations for forward reactions. Bold fonts denote optimal conformations.

Target	Ligand		Hartree energy for target-ligand complex		Hartree energy for ligand in solution		Hartree Energy for ΔG = Δcomplex - Δligand		ΔGj - ΔGi		Ki ligand I / Ki ligand j	
	i	j	i	j	i	j	i	j	kcal/mol	kJ/mol	calculated	experimental
**CDK2**	**1**	**3**	**-501.38112**	**-504.54515**	**-51.53004**	**-54.69374**	**-449.85108**	**-449.85141**	**-0.21**	**-0.87**	**1.4**	**0.6**
**CDK2**	1	4_sin	-501.38112	-501.65263	-51.53004	-51.80246	-449.85108	-449.85017	0.57	2.39	0.4	2.7
**CDK2**	**1**	**4_anti**	**-501.38112**	**-501.65909**	**-51.53004**	**-51.80390**	**-449.85108**	**-449.85519**	**-2.58**	**-10.79**	**77.7**	**2.7**
**CDK2**	1	2_anti	-501.38112	-505.45764	-51.53004	-55.59894	-449.85108	-449.85870	-4.78	-20.01	3200.0	4.4
**CDK2**	**1**	**2_sin**	**-501.38112**	**-505.46070**	**-51.53004**	**-55.61029**	**-449.85108**	**-449.85041**	**0.42**	**1.76**	**0.5**	**4.4**
**CDK1**	**1**	**3**	**-582.05086**	**-585.21235**	**-51.52890**	**-54.69075**	**-530.52196**	**-530.52160**	**0.23**	**0.95**	**0.7**	**0.4**
**CDK1**	1	4_sin	-582.05086	-582.32137	-51.52890	-51.80149	-530.52196	-530.51988	1.31	5.46	0.1	2.2
**CDK1**	**1**	**4_anti**	**-582.05086**	**-582.32556**	**-51.52890**	**-51.80324**	**-530.52196**	**-530.52232**	**-0.23**	**-0.95**	**1.5**	**2.2**
**CDK1**	1	2_anti	-582.05086	-586.12765	-51.52890	-55.60141	-530.52196	-530.52624	-2.69	-11.24	93.1	5.4
**CDK1**	**1**	**2_sin**	**-582.05086**	**-586.12955**	**-51.52890**	**-55.60906**	**-530.52196**	**-530.52049**	**0.92**	**3.86**	**0.2**	**5.4**
**CDK9**	**1**	**3**	**-625.09413**	**-628.25610**	**-51.53226**	**-54.69516**	**-573.56187**	**-573.56094**	**0.58**	**2.44**	**0.4**	**0.7**
**CDK9**	1	4_sin	-625.09413	-625.36439	-51.53226	-51.80254	-573.56187	-573.56185	0.01	0.05	1.0	3.5
**CDK9**	**1**	**4_anti**	**-625.09413**	**-625.37052**	**-51.53226**	**-51.80696**	**-573.56187**	**-573.56356**	**-1.06**	**-4.44**	**6.0**	**3.5**
**CDK9**	1	2_anti	-625.09413	-629.16950	-51.53226	-55.60447	-573.56187	-573.56503	-1.98	-8.30	28.5	2.7
**CDK9**	**1**	**2_sin**	**-625.09413**	**-629.17478**	**-51.53226**	**-55.61284**	**-573.56187**	**-573.56194**	**-0.04**	**-0.18**	**1.1**	**2.7**
**CDK5**	**1**	**3**	**-516.84348**	**-520.00231**	**-51.53030**	**-54.69280**	**-465.31318**	**-465.30951**	**2.30**	**9.64**	**0.02**	**1**
**CDK5**	**1**	**4_sin**	**-516.84348**	**-517.11323**	**-51.53030**	**-51.80363**	**-465.31318**	**-465.30960**	**2.25**	**9.40**	**0.02**	**1.6**
**CDK5**	1	4_anti	-516.84348	-517.10979	-51.53030	-51.80492	-465.31318	-465.30487	5.21	21.82	0.0	1.6
**CDK5**	**1**	**2_anti**	**-516.84348**	**-520.92255**	**-51.53030**	**-55.60305**	**-465.31318**	**-465.31950**	**-3.97**	**-16.59**	**807.5**	**35.5**
**CDK5**	1	2_sin	-516.84348	-520.92207	-51.53030	-55.60371	-465.31318	-465.31836	-3.25	-13.60	241.4	35.5

Table S5. QM-based ΔΔG calculations for reverse reactions. Bold fonts denote optimal conformations.

**Target**	**Ligand**		**Hartree energy for target-ligand complex**		**Hartree energy for ligand in solution**		**Hartree Energy for ΔG = Δcomplex - Δligand**		**ΔGj - ΔGi**		**Ki $^{ligand\ I}$ / Ki $^{ligand\ j}$**	
	**j**	**i**	**j**	**i**	**j**	**i**	**j**	**i**	**kcal/mol**	**kJ/mol**	**calculated**	**experimental**
**CDK2**	**3**	**1**	**-504.54515**	**-501.38108**	**-54.69374**	**-51.52993**	**-449.85141**	**-449.85115**	**-0.16**	**-0.68**	**1.3**	**0.6**
**CDK2**	4_sin	1	-501.65263	-501.38111	-51.80246	-51.53004	-449.85017	-449.85107	0.56	2.36	0.4	2.7
**CDK2**	**4_anti**	**1**	**-501.65909**	**-501.38112**	**-51.80390**	**-51.53005**	**-449.85519**	**-449.85107**	**-2.59**	**-10.82**	**78.6**	**2.7**
**CDK2**	2_anti	1	-505.45764	-501.38112	-55.59894	-51.52996	-449.85870	-449.85116	-4.73	-19.80	2940.0	4.4
**CDK2**	**2_sin**	**1**	**-505.46070**	**-501.38111**	**-55.61029**	**-51.53004**	**-449.85041**	**-449.85107**	**0.41**	**1.73**	**0.5**	**4.4**
**CDK1**	**3**	**1**	**-585.21235**	**-582.05106**	**-54.69075**	**-51.52863**	**-530.52160**	**-530.52243**	**0.52**	**2.18**	**0.4**	**0.4**
**CDK1**	4_sin	1	-582.32137	-582.05050	-51.80149	-51.52831	-530.51988	-530.52219	1.45	6.06	0.1	2.2
**CDK1**	**4_anti**	**1**	**-582.32556**	**-582.05003**	**-51.80324**	**-51.52792**	**-530.52232**	**-530.52211**	**-0.13**	**-0.55**	**1.2**	**2.2**
**CDK1**	2_anti	1	-586.12765	-582.05085	-55.60141	-51.52880	-530.52624	-530.52205	-2.63	-11.00	84.6	5.4
**CDK1**	**2_sin**	**1**	**-586.12955**	**-582.04990**	**-55.60906**	**-51.52804**	**-530.52049**	**-530.52186**	**0.86**	**3.60**	**0.2**	**5.4**
**CDK9**	**3**	**1**	**-628.25610**	**-625.09409**	**-54.69516**	**-51.53234**	**-573.56094**	**-573.56175**	**0.51**	**2.13**	**0.4**	**0.7**
**CDK9**	4_sin	1	-625.36439	-625.09414	-51.80254	-51.53218	-573.56185	-573.56196	0.07	0.29	0.9	3.5
**CDK9**	**4_anti**	**1**	**-625.37052**	**-625.09422**	**-51.80696**	**-51.53233**	**-573.56356**	**-573.56189**	**-1.05**	**-4.38**	**5.9**	**3.5**
**CDK9**	2_anti	1	-629.16950	-625.09417	-55.60447	-51.53223	-573.56503	-573.56194	-1.94	-8.11	26.4	2.7
**CDK9**	**2_sin**	**1**	**-629.17478**	**-**	**-55.61284**	**-51.53211**	**-**	**-573.56203**	**0.06**	**0.24**	**0.9**	**2.7**

				**625.09414**			**573.56194**					
**CDK5**	**3**	**1**	**-520.00231**	**-516.84341**	**-54.69280**	**-51.53034**	**-465.30951**	**-465.31307**	**2.23**	**9.35**	**0.02**	**1**
**CDK5**	**4_sin**	**1**	**-517.11323**	**-516.84338**	**-51.80363**	**-51.53033**	**-465.30960**	**-465.31305**	**2.16**	**9.06**	**0.03**	**1.6**
**CDK5**	4_anti	1	-517.10979	-516.84349	-51.80492	-51.53029	-465.30487	-465.31320	5.23	21.87	0.0	1.6
**CDK5**	**2_anti**	**1**	**-520.92255**	**-516.84349**	**-55.60305**	**-51.53028**	**-465.31950**	**-465.31321**	**-3.95**	**-16.51**	**782.3**	**35.5**
**CDK5**	2_sin	1	-520.92207	-516.84342	-55.60371	-51.53035	-465.31836	-465.31307	-3.32	-13.89	271.2	35.5

1.2.2.1 Correlation with experimental data

This method allows for the prediction of binding energies and biological activity of inhibitors even when 3D structure of the complex is unknown. The correlation coefficient R^2 between experimental and calculated data was 0.9681. Although the precision is compromised when the structure of the complex is unknown, the method provides a reasonably accurate prediction of the trend of the biological activity of chemically similar ligands (Figure S1).

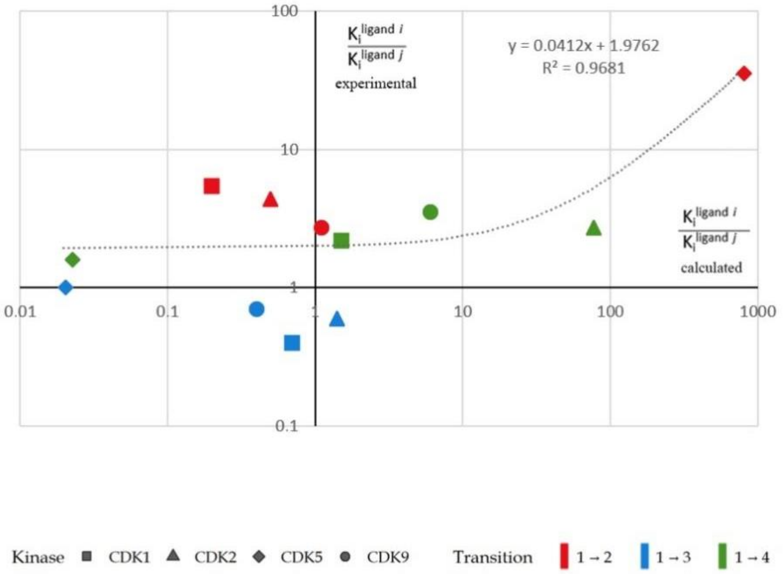

Figure S1. Correlation analyses for experimental and calculated ratios of inhibition constants in the log scale. The dashed line shows the identity.

1.3 MD and NEQ thermodynamics

The calculation of the free energy difference between ligands A and B included ligand and protein preparation, generation of protein-ligand complexes, equilibrium and NEQ thermodynamics. The ligand preparation procedure included parameterization of ligands A and B and generation of a hybrid structure used to obtain solvated ligands and ligand-protein complexes. The resulting systems with ligands A and B in bound and unbound states were equilibrated for 1,000 ps. Then, 10 ns MD was performed to generate 100 trajectory frames that were used for ΔΔG calculations by NEQ thermodynamics (Figure S2).

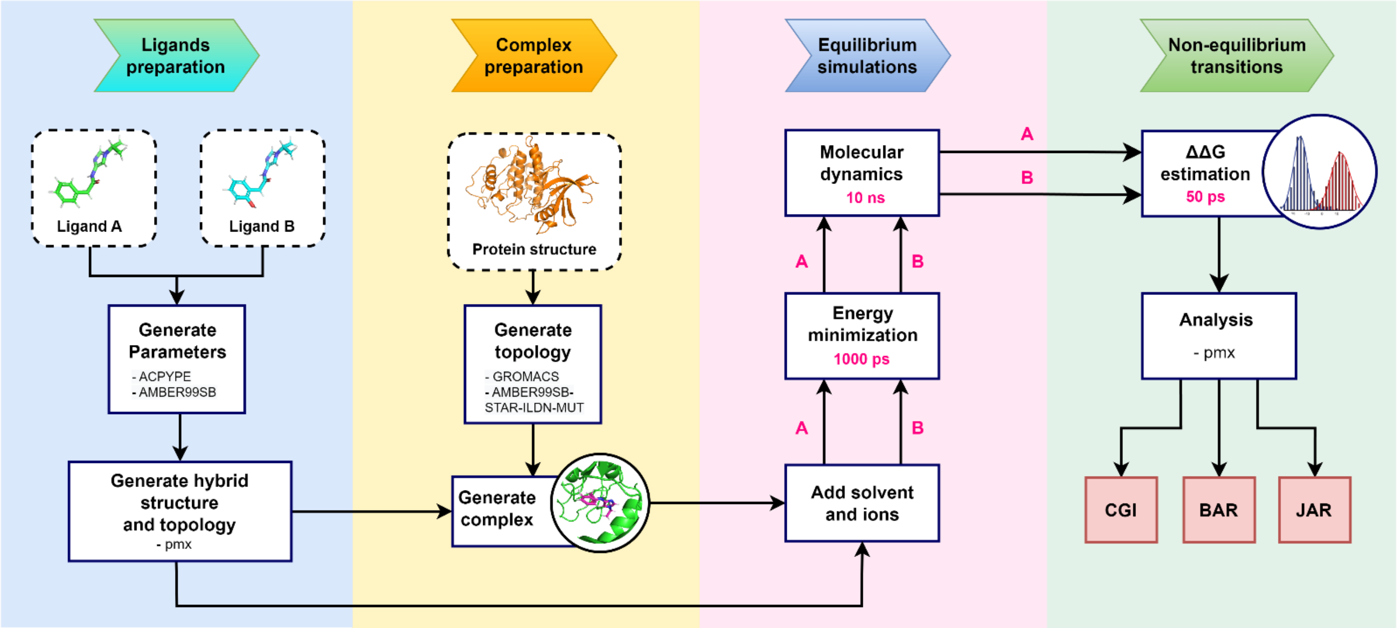

Figure S2. The computational algorithm for ΔΔG calculation based on pmx protocol [8].

1.3.1 Analysis of key integrations using MD trajectories

Table S6. Key interactions for OH group in **2** and N atom in **4** in complexes with CDK1, -2, -5, and- 9 obtained by MD trajectory analysis.

<table>
<tr><td rowspan="2">Target</td><td colspan="2">Conformation</td></tr>
<tr><td>sin-</td><td>anti-</td></tr>
<tr><td rowspan="2">CDK2</td><td>ligand 2
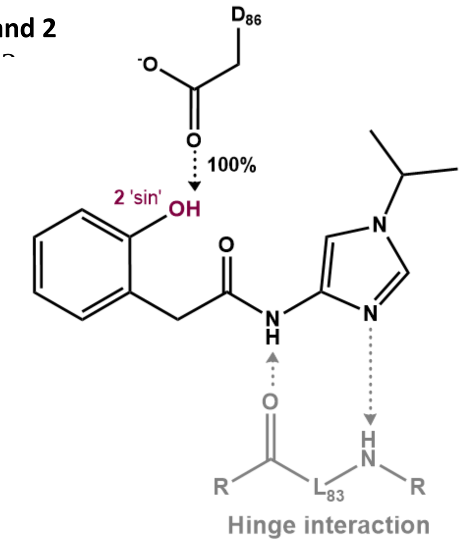
</td><td>ligand 2
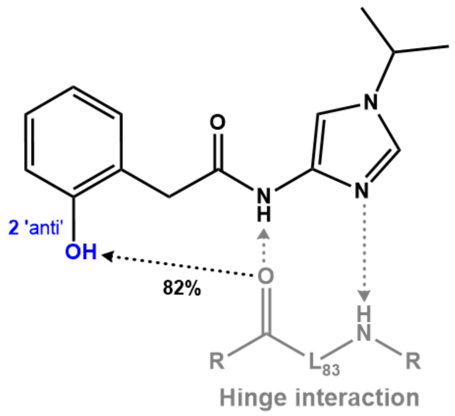
</td></tr>
<tr><td>ligand 4
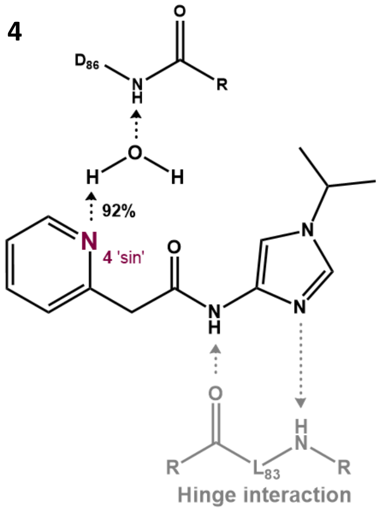
</td><td>ligand 4
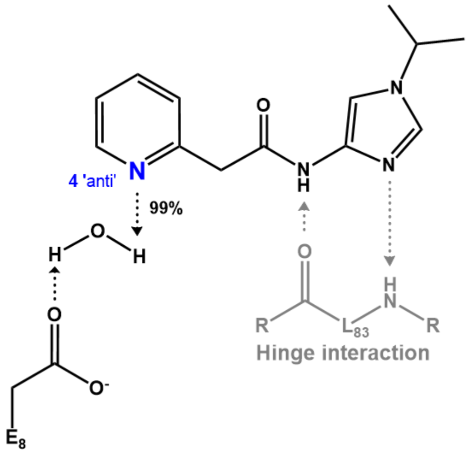
</td></tr>
</table>

CDK1	**ligand 2**<br>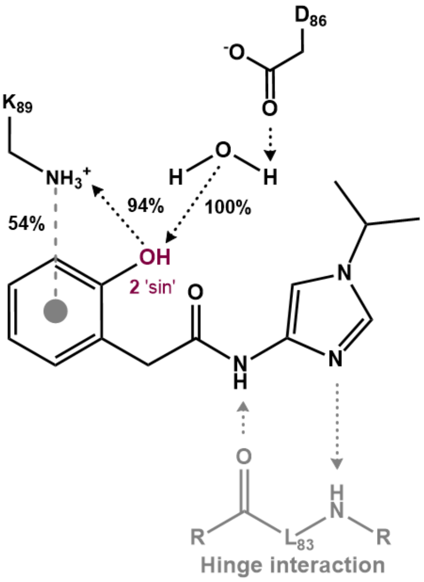	**ligand 2**<br>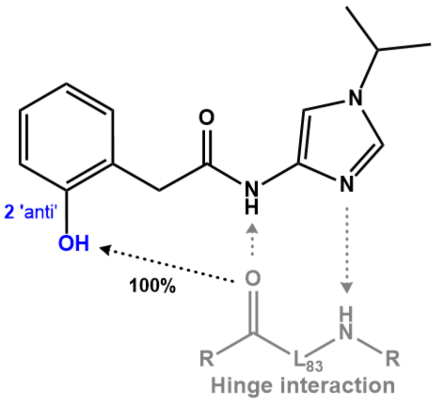
	**ligand 4**<br>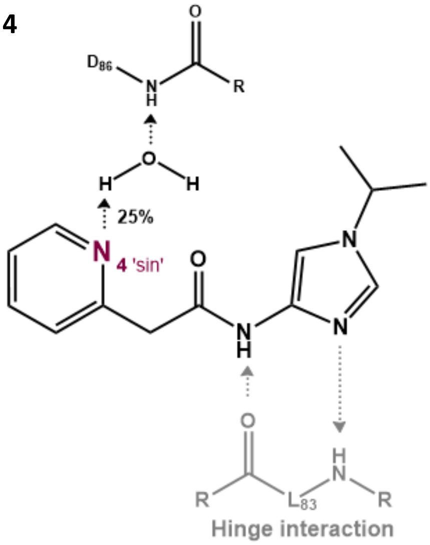	**ligand 4**<br>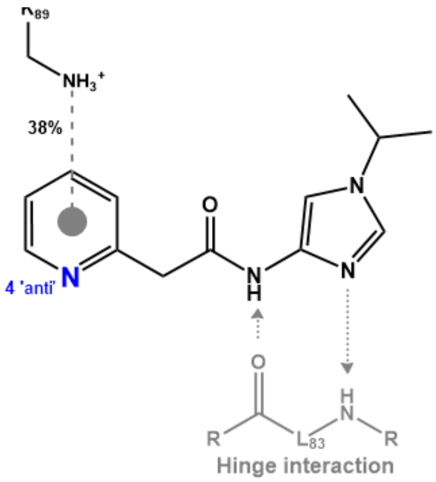

CDK5	**ligand 2** 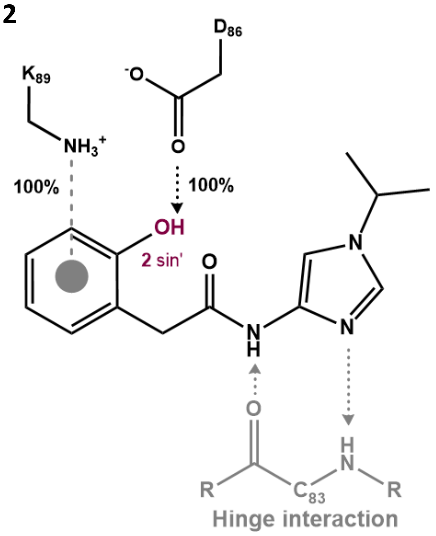	**ligand 2** 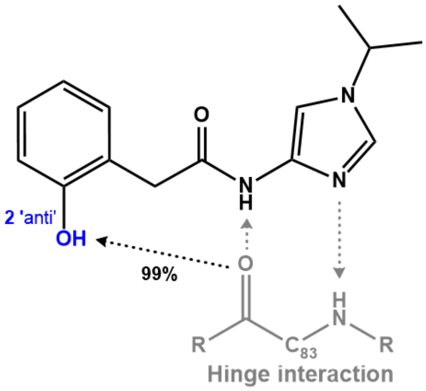
	**ligand 4** 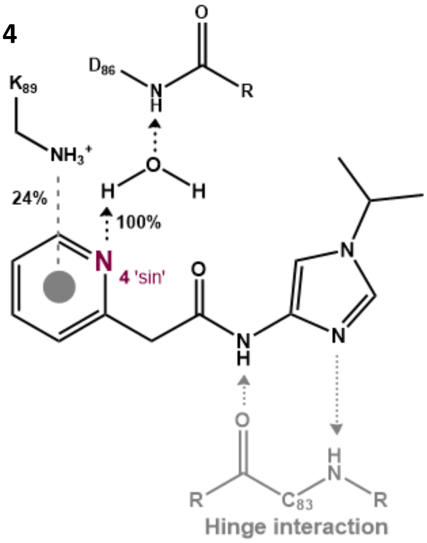	**ligand 4** 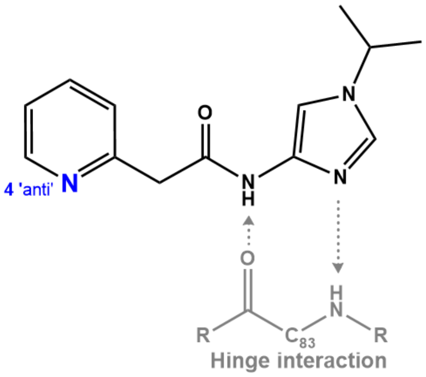

CDK9	**ligand 2** 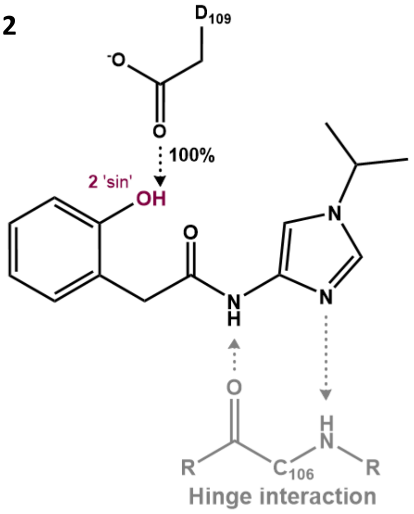	**ligand 2** 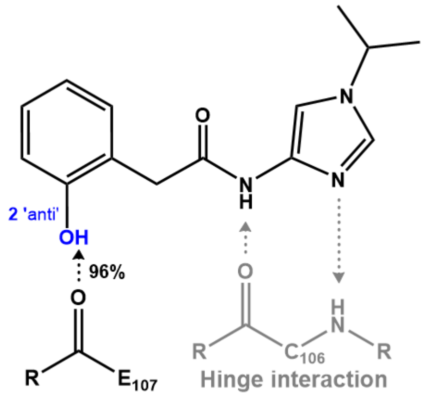
	**ligand 4** 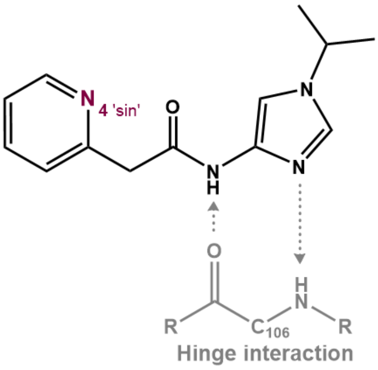	**ligand 4** 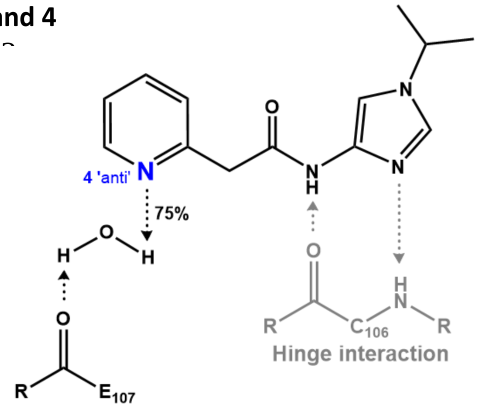

1.3.2 Correlation with experimental data

Table S7. ΔΔG calculations. Green indicates ddG values that differ significantly from zero; gray indicates an unreliable difference from zero. Red indicates an incorrect forecast. Bold in the Transition column denotes conformations that correspond to energy minima.

Target	Transition	ΔΔG(exp) kJ/mol			ΔΔG(CGI) kJ/mol			ΔΔG(BAR) kJ/mol			ΔΔG(JAR) kJ/mol		
**CDK2**	**1 → 2 sin**	-3.7	±	0.3	-7.9	±	1.8	-12.4	±	0.9	-12.7	±	1.4
**CDK2**	1 → 2 anti	-3.7	±	0.3	0.9	±	2.4	0.1	±	2.2	4.1	±	2.1
**CDK2**	**1 → 3**	1.2	±	0.2	-0.8	±	0.9	-3.1	±	0.7	2.2	±	1.2
**CDK2**	**1 → 4 sin**	-2.3	±	0.2	-3.5	±	1.1	-4.8	±	1.0	-5.7	±	1.6
**CDK2**	1 → 4 anti	-2.3	±	0.2	1.2	±	2.2	-3.9	±	1.6	-4.5	±	1.8
**CDK1**	**1 → 2 sin**	-7.3	±	0.9	-6.4	±	1.4	-6.4	±	1.3	-7.3	±	2.0
**CDK1**	1 → 2 anti	-7.3	±	0.9	15.1	±	1.0	15.6	±	0.8	14.1	±	1.3
**CDK1**	**1 → 3**	1.8	±	1.0	2.1	±	1.5	0.5	±	1.1	1.8	±	2.0
**CDK1**	1 → 4 sin	-2.0	±	0.9	-1.4	±	1.5	0.5	±	1.8	2.7	±	3.2
**CDK1**	**1 → 4 anti**	-2.0	±	0.9	-8.5	±	2.0	-4.5	±	2.0	2.8	±	5.0
**CDK5**	**1 → 2 sin**	-9.3	±	0.7	-22.7	±	1.1	-21.6	±	0.9	-20.9	±	2.2
**CDK5**	1 → 2 anti	-9.3	±	0.7	8.9	±	2.0	10.7	±	1.1	10.7	±	1.4
**CDK5**	**1 → 3**	1.7	±	0.8	4.0	±	1.8	4.0	±	2.4	2.9	±	2.7
**CDK5**	**1 → 4 sin**	-1.7	±	0.5	-3.4	±	1.6	-2.8	±	2.3	-3.1	±	3.2
**CDK5**	1 → 4 anti	-1.7	±	0.5	2.1	±	1.7	3.0	±	1.9	4.2	±	2.9
**CDK9**	**1 → 2 sin**	-2.6	±	0.9	-2.3	±	1.8	-1.5	±	0.8	-1.8	±	1.1
**CDK9**	1 → 2 anti	-2.6	±	0.9	10.0	±	2.7	7.3	±	1.3	9.3	±	1.3
**CDK9**	**1 → 3**	0.9	±	0.8	6.3	±	1.4	7.0	±	1.5	6.2	±	3.6
**CDK9**	1 → 4 sin	-3.0	±	0.6	9.8	±	1.3	10.9	±	1.6	11.6	±	2.7
**CDK9**	**1 → 4 anti**	-3.0	±	0.6	-2.0	±	1.3	-1.1	±	1.0	-0.9	±	1.3

Table S8. ΔS calculations. Color codes: green, ΔS differs significantly from zero; gray, difference from zero is not reliable. Pink, incorrect prediction. Bold in the Transition column denotes conformations that correspond to energy minima.

Target	**Transition**	**ΔS(exp) kJ/mol**			**ΔS(CGI) kJ/mol**			**ΔS(BAR) kJ/mol**			**ΔS(JAR) kJ/mol**		
**CDK1**	**1 → 2 sin**	-3.6	±	1.2	1.5	±	3.2	-6.4	±	2.2	5.4	±	3.4
CDK1	1 → 2 anti	-3.6	±	1.2	6.7	±	2.6	6.2	±	2.0	8.2	±	6.2
**CDK1**	**1 → 3**	0.6	±	1.2	2.9	±	2.4	3.6	±	1.8	-0.4	±	3.2
CDK1	1 → 4 sin	0.3	±	1.2	-2.6	±	3.7	4.4	±	3.4	7.2	±	5.0
**CDK1**	**1 → 4 anti**	0.3	±	1.2	-5.0	±	3.1	0.3	±	3.0	8.5	±	6.6
**CDK5**	**1 → 2 sin**	-5.6	±	1.0	-14.8	±	2.8	-21.6	±	1.8	-8.2	±	3.7
CDK5	1 → 2 anti	-5.6	±	1.0	0.6	±	3.7	1.3	±	2.3	4.8	±	6.3
**CDK5**	**1 → 3**	0.5	±	1.0	4.9	±	2.8	7.1	±	3.0	0.7	±	3.9
**CDK5**	**1 → 4 sin**	0.7	±	0.8	0.1	±	2.8	2.1	±	3.3	2.7	±	4.7
CDK5	1 → 4 anti	0.7	±	0.8	1.0	±	3.8	6.9	±	3.4	8.7	±	4.8
**CDK9**	**1 → 2 sin**	1.1	±	1.2	5.6	±	3.5	-1.5	±	1.7	10.9	±	2.5
CDK9	1 → 2 anti	1.1	±	1.2	1.6	±	4.4	-2.1	±	2.4	3.4	±	6.3
**CDK9**	**1 → 3**	-0.4	±	1.0	7.1	±	2.3	10.2	±	2.2	3.9	±	4.7
CDK9	1 → 4 sin	-0.7	±	0.8	8.6	±	3.5	14.8	±	3.1	16.1	±	4.5
**CDK9**	**1 → 4 anti**	-0.7	±	0.8	1.4	±	2.4	3.8	±	2.0	4.8	±	2.9

2 Calculations of conformational focusing energy

For compounds **1, 2** and **4** a relaxed potential energy surface scan of O=C-C-C and C-C-C-C torsion angles were carried out at PBE0 [51]-D3BJ [52, 53]/def2-SVP [54] level of theory at ambient temperature in 36 steps with 10° growth using Gaussian16 A.03 [55]. Water solvation effects were included using a polarizable continuum model [56]; quasi-harmonic corrections for free energies were computed using GoodVibes [57]. For **2** and **4** the minima with H- bonds were found (Table S6, Figure S3). For the latter compound a water molecule was added to the system to allow H-bonds.

Compound **4** is capable of forming multiple sets of H-bonds depending on the torsion angle. While there is a mandatory H-bond between water molecule and nitrogen in pyridine, another possible bond may be formed between water and either carbonyl oxygen or amide hydrogen or even imidazole ring. Otherwise water can form not the additional bonds but instead bind to other H_2O molecules. Binding to the amide hydrogen is energetically the most favorable (99.7% contribution). Compound **2** exists in the sole possible conformation with the H-bond while **1** and **3** lack significant conformational energy barriers and are presented as a number of potential conformations. Regarding the orientation of the imidazole ring relative to the amide group, we found that the amide oxygen and imidazole hydrogen were in *cis* configuration.

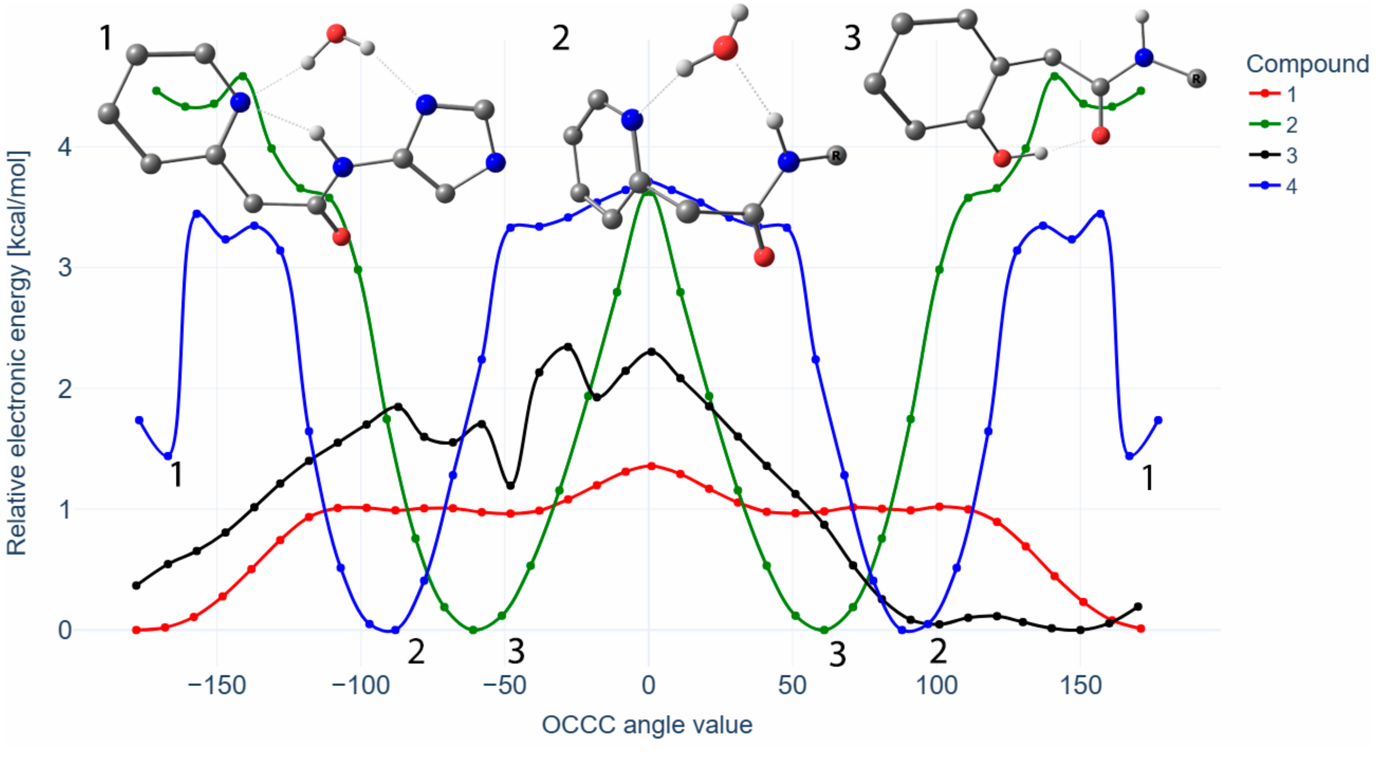

Figure S3. Correlation between relative electronic energy and OCCC angles for **1-4**.

Shown are the values relative to the lowest energy conformation of the respective compound, therefore individual structures are incomparable. The 3D structures correspond to energy minimums with H-bonds.

For better presentation of conformational focusing, we drew the contour maps of 2D potential energy surfaces of **1** and **3** formed by scanning along O=C-C-C and C-C-C-C torsion angles (Figure S4). On top of these maps we

overlaid the points from MD of the corresponding compounds in CDK2. From these plots one can conclude that compound **3** is more labile in water than **1** by ~ 31% (12.6% *vs* 9.6% points on the map are within 1 kcal/mol from minimum). Therefore **3** adopts more conformations. Both compounds exist in water and in CDK2 in different forms making the conformational focusing a critical requirement for drug-complex formation.

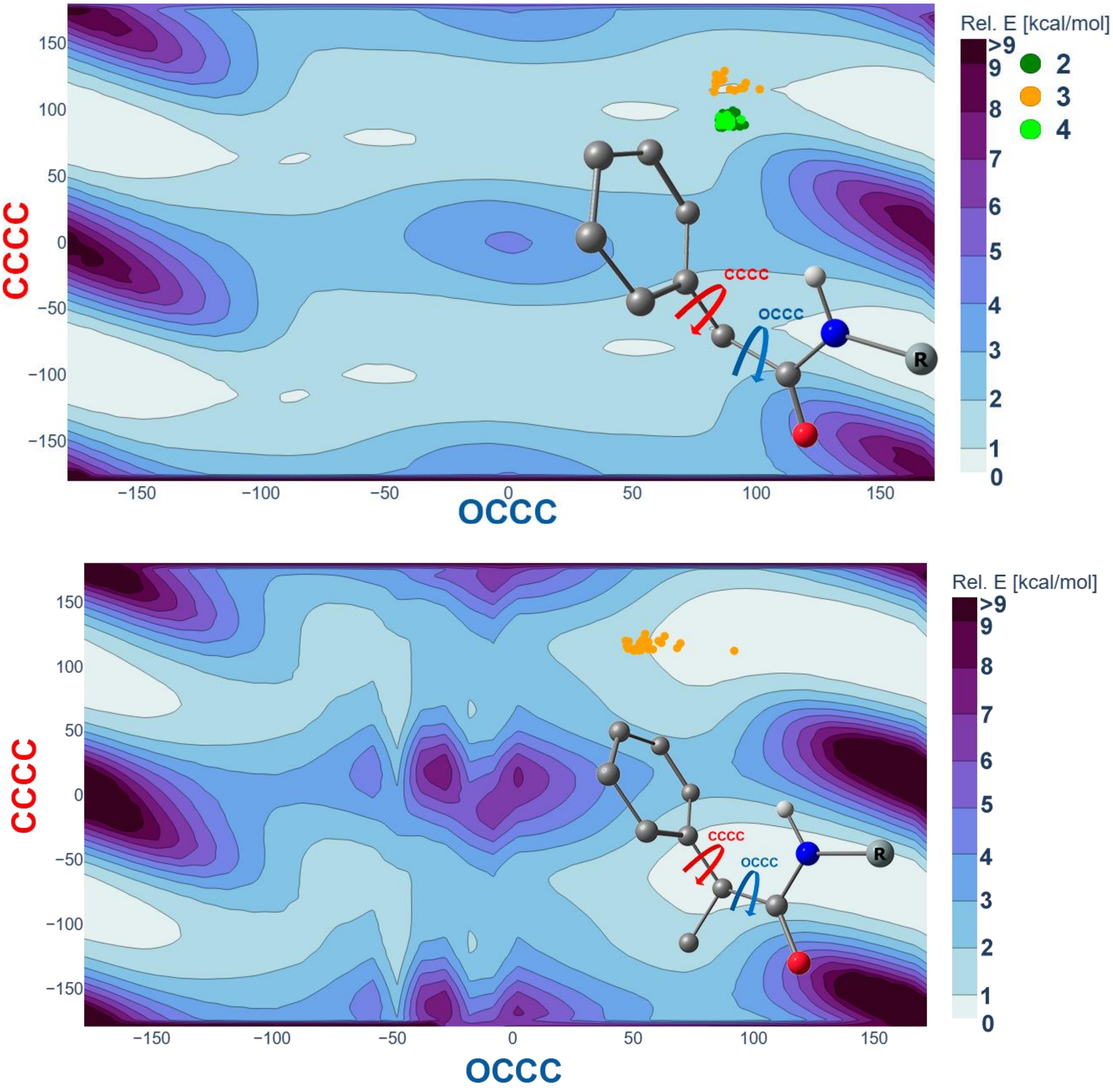

Figure S4. The contour map of 2D potential energy surfaces of **1** (top) and **3** (bottom) with overlaid points based on MD.

3 Calculations of total electrostatic potential

Total electrostatic potential is used to analyze the positively and negatively charged regions of a molecule. It is defined as the electrostatic interaction energy of the molecule with an infinitesimal positive point charge. To evaluate the stacking properties of **1-4** we analyzed the total electrostatic potential of aromatic rings using Multiwfn software [58, 59]. Dark red regions on the isosurfaces (Figure S5) indicate strong negative charges suggesting bigger energies of interaction of **2** and **4** with the positively charged Lys residue compared to **1** and **3**. In case of **4** there is no stacking; the nitrogen atom is oriented towards the cation.

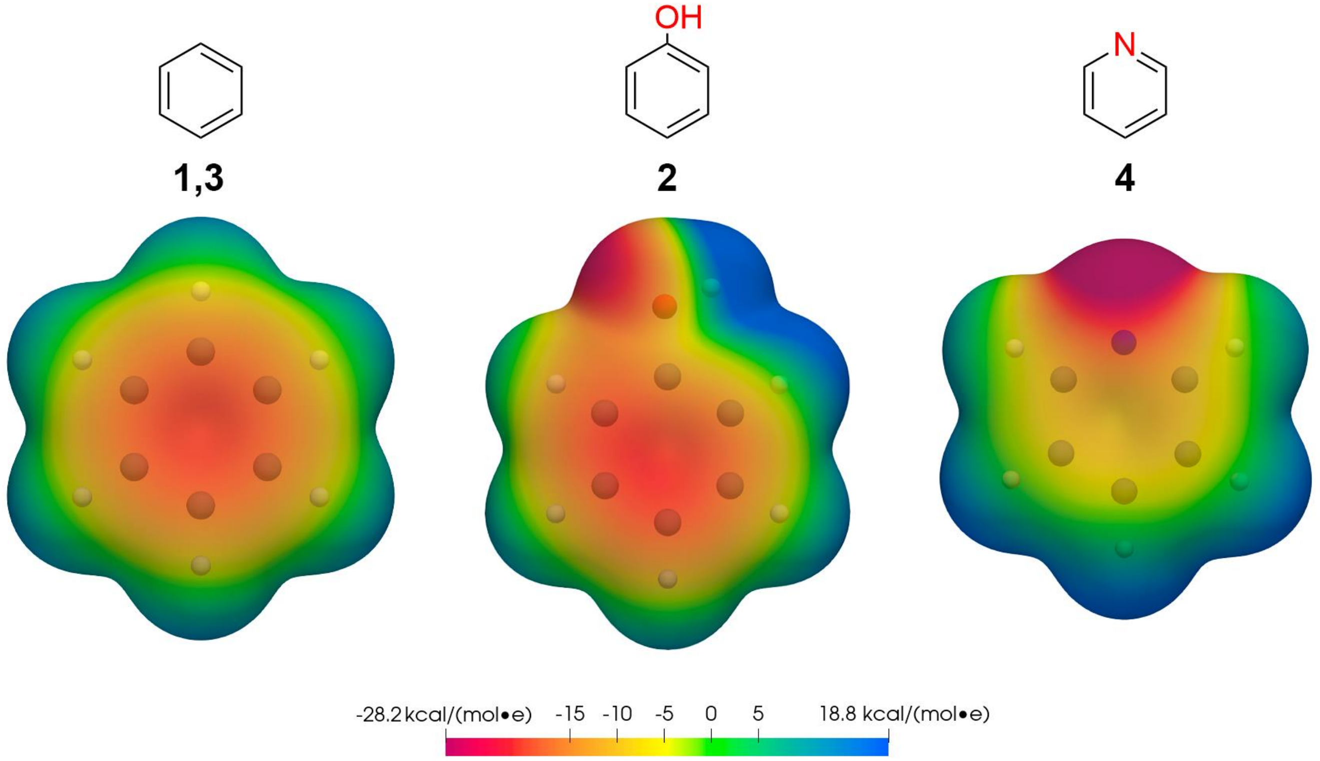

Figure S5. Total electrostatic potentials for arenes.

Numbers indicate the compounds with the corresponding fragments.
